# Genome wide association analysis of the heart using high-resolution 3D cardiac MRI identifies new genetic loci underlying cardiac structure and function

**DOI:** 10.1186/1532-429X-18-S1-Q63

**Published:** 2016-01-27

**Authors:** Antonio de Marvao, Hannah V Meyer, Timothy J Dawes, Wenzhe Shi, Wenjia Bai, Daniel Rueckert, Ewan Birney, Declan P O'Regan, Stuart Cook

**Affiliations:** 1 grid.7445.20000000121138111Medical Research Council Clinical Sciences Centre, Imperial College London, London, United Kingdom; 2 grid.225360.00000000097097726European Bioinformatics Institute, European Molecular Biology Laboratory, Cambridge, United Kingdom; 3 grid.7445.20000000121138111Department of Computing, Imperial College London, London, United Kingdom; 4 grid.428397.30000000403850924Duke-NUS Graduate Medical School, Singapore, Singapore

## Background

Human physiology and disease are defined by complex interactions between genetic and environmental factors. While genome-wide association studies (GWASs) have been successfully used to dissect many human traits, they have not worked for studies of the heart. This likely reflects the limitations in echocardiographic and traditional magnetic resonance phenotyping, as many cardiac traits are known to be highly heritable. In an attempt to overcome the limitations of human heart GWASs to date, we developed high-resolution 3D-cardiac MRI (3D-CMR) techniques combined with computational image analysis and dense genotyping to examine the common genetic determinants of cardiac structure and function.

## Methods

Healthy volunteers (n = 1850; 55% females, mean age 41 ± 13 years) were recruited prospectively to the Digital Heart Project and underwent both standard CMR and high-spatial resolution 3D-CMR (Philips 1.5T Achieva, Best, Netherlands). Using a cardiac atlas-based software, 3D-CMR images were computationally processed and quantitatively analysed. Anatomical and functional parameters including left ventricular shape, curvature, wall thickness, wall stress, fractional wall thickening, mass and ventricular volumes were extracted at over 46,000 points in each model. The 2D datasets were analysed using commercially available software. Subjects were genotyped using a standard SNP array and genotypes were imputed against a combined UK10K / 1000 Genomes reference panel, after which 9.4 million variants were available for the GWAS. Bayesian latent factor analysis was used to reduce high dimensional 3D morphological and functional phenotypes into an informative lower dimensional space (n = 100 factors). These factors were used for single trait linear mixed model association analysis. 2D metrics such as LV mass, volumes and ejection fraction were also subjected to GWAS.

## Results

**Figure 1:**
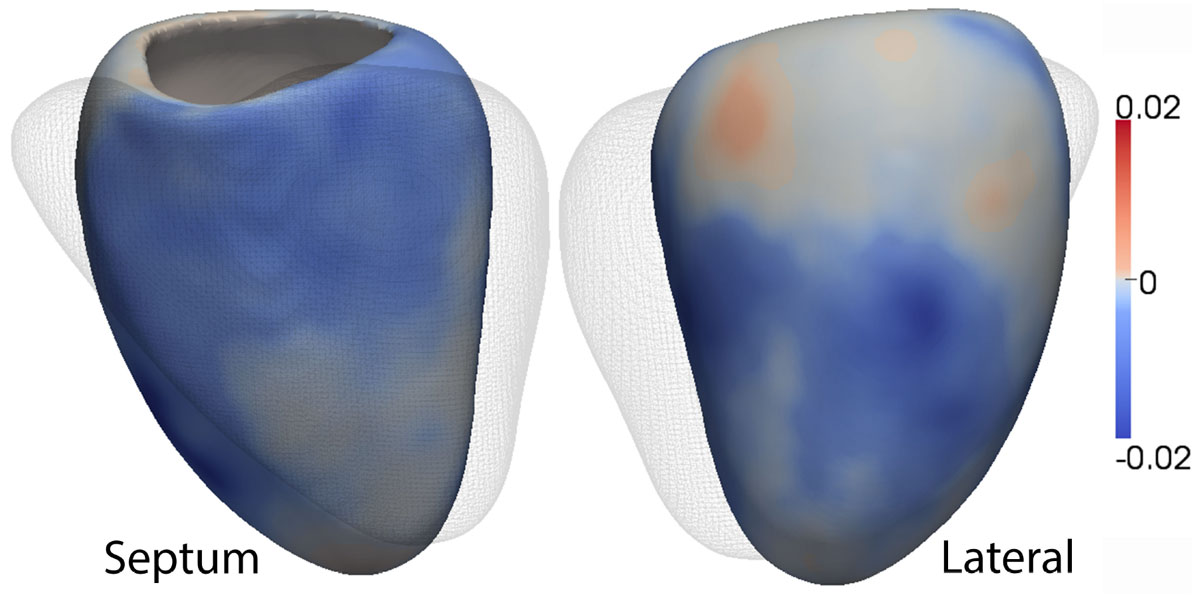
**A 3D model of the left ventricle illustrating the strongest association between a SNP in the genome wide association study and a Bayesian factor projection of wall thickness**. The linear mixed model regression coefficients at each point are plotted in the epicardial surface of the left ventricle with the right ventricle depicted as a mesh. This SNP is negatively associated with wall thickness in the basal and mid septal regions and positively associated with wall thickness in the basal anterolateral area.

A large number (n>10) of genome-wide significant loci were identified, which demonstrated strong associations with regional differences in LV wall thickness and function. These regions are located in contiguous and biologically plausible areas of the ventricle (Figure 1). Several of the top associations were found at loci encoding important cardiac development genes. In contrast, there were few significant associations uncovered by GWAS of the 2D data.

## Conclusions

3D-CMR combined with computational modeling and dense genotyping provides novel and high-resolution insights into the effects of genetic factors on the heart. Our data demonstrates that common genetic variation is associated with regional rather than global changes in cardiac morphology and function, which would be missed using traditional CMR- or echocardiography-derived phenotypes, and reveals novel genes underlying cardiac physiology.

